# Cerebrospinal Fluid Drop Metastases of Canine Glioma: Magnetic Resonance Imaging Classification

**DOI:** 10.3389/fvets.2021.650320

**Published:** 2021-05-03

**Authors:** R. Timothy Bentley, Amy B. Yanke, Margaret A. Miller, Hock Gan Heng, Aaron Cohen-Gadol, John H. Rossmeisl

**Affiliations:** ^1^Department of Veterinary Clinical Sciences, College of Veterinary Medicine, Purdue University, West Lafayette, IN, United States; ^2^Department of Clinical Sciences, College of Veterinary Medicine, Auburn University, Auburn, AL, United States; ^3^Department of Comparative Pathobiology, College of Veterinary Medicine, Purdue University, West Lafayette, IN, United States; ^4^Department of Neurological Surgery, Indiana University School of Medicine, Indianapolis, IN, United States; ^5^Department of Small Animal Clinical Sciences, Virginia-Maryland College of Veterinary Medicine, Blacksburg, VA, United States

**Keywords:** astrocytoma, dissemination, dog, ependyma, glioblastoma, leptomeninges, meninges, oligodendroglioma

## Abstract

Dissemination of glioma in humans can occur as leptomeningeal nodules, diffuse leptomeningeal lesions, or ependymal lesions. Cerebrospinal fluid (CSF) drop metastasis of glioma is not well-recognized in dogs. Ten dogs with at least two anatomically distinct and histologically confirmed foci of glioma were included in this study. The 10 dogs underwent 28 magnetic resonance imaging (MRI) examinations, with distant CSF drop metastasis revealed in 13 MRIs. The CSF drop metastases appeared as leptomeningeal nodules in four dogs, diffuse leptomeningeal lesions in six dogs, and ependymal lesions in seven dogs; six dogs had a combination of lesion types. Primary tumors were generally T2-heterogeneous and contrast-enhancing. Many metastases were T2-homogeneous and non-enhancing. Diffuse leptomeningeal lesions were seen as widespread extra-axial contrast-enhancement, again very dissimilar to the intra-axial primary mass. Primary masses were rostrotentorial, whereas metastases generally occurred in the direction of CSF flow, in ventricles, CSF cisterns, and the central canal or leptomeninges of the cervical or thoracolumbar spinal cord. Seven of the dogs had received therapy limited to the primary mass, such as surgery or stereotactic radiation, then developed metastasis in the following months. CSF drop metastasis of glioma may take a very different appearance on MRI to the primary mass, including periventricular lesions that are more homogeneous and less contrast-enhancing, rostral horn signal changes, or leptomeningeal enhancement ventral to the brainstem or encircling the spinal cord.

## Introduction

The magnetic resonance imaging (MRI) features of canine gliomas have been described, but most studies focus upon a solitary mass (1–3). Oligodendroglioma, in particular, may occur near the cerebrospinal fluid (CSF) in peri-ventricular or even intra-ventricular locations (3, 4). As we prolong the survival of increasing numbers of dogs with surgery, radiation, and other interventions, we are beginning to appreciate more glioma metastases. An increasing metastatic caseload was similarly observed in people (5). Two canine case reports describe oligodendroglioma disseminating *via* the CSF, a process known as “CSF drop metastasis (6, 7).” Such metastasis often occurs in a caudal direction, in accordance with CSF flow. In both dogs, a solitary forebrain mass was initially treated with radiation. After treatment, sequential metastatic lesions were seen on follow-up MRI, first more caudally within the brain and then in the cervical spinal cord (6, 7). Neurological deficits can progress from focal and forebrain to vestibular, cervical, or multifocal (6, 7). In other canine oligodendrogliomas, CSF drop metastasis has been suspected at first presentation (8).

The MRI characteristics of canine glioma CSF drop metastasis are not well-described. A scheme exists for classifying the dissemination of human glioma (5). The goal of this retrospective multi-institutional study was, therefore, to describe the MRI findings of canine glioma CSF drop metastasis and categorize them according to the human classification system (5). Based on the two canine case reports (6, 7), we hypothesized that the metastatic lesions would have different imaging characteristics to the primary tumor.

## Results

### Signalment and Clinical History

Six male (four castrated) and four spayed female dogs, aged 2.3-12.9 (median, 6.7) years, met the inclusion criteria. This included three Boxer dogs, two English Bulldogs, and one each of French Bulldog, Staffordshire Terrier, American Pit Bull Terrier mix, Border Terrier, and Dachshund.

We recognized two clinically distinct groups of cases (Table 1). The first group of gliomas initially presented as solitary lesions, underwent various therapies and later metastasized (Group A; *n* = 7). Group B cases had multifocal lesions (primary and metastatic) at first presentation (*n* = 3).

**Table 1:** Magnetic resonance imaging of cerebrospinal fluid drop metastases in canine glioma.

**Case**	**Primary Mass**	**Type Ia Leptomeningeal nodules**	**Type 1b Diffuse leptomeningeal lesions**	**Type II Ependymal Lesions**	**Type IV Mixed**
**A1** 6.3-year M Boxer	IA—periventricular (LV) HGO		Midbrain to cervical spinal cord	5 lesions including 1 nodule (LVs, V3, V4)	Yes
**A2** 4.5-year MN Pit Bull-Mix	IA—periventricular (LV) HGO			2 nodules (V4 and LV)	No
**A3** 9.0-year MN Border	IA—periventricular (LV) O	1 nodule (cerebral)			No
**A4** 12.9-year FS Dachshund	SAS—optic chiasm HGA		Optic tracts and parasellar region	Periventricular hyperintensities (LVs, V3, aqueduct)	Yes
**A5** 11.0-year MN Staff Terrier	IA—periventricular (LV) HGA	5 nodules (cerebral)		1 lesion (LV)	Yes
**A6** 2.3-year MN Eng Bulldog	IA bulging into LV HGO			6 nodules (LVs) Extensive ependymal enhancement (LVs)	No
**A7** 7.1-year M Eng Bulldog	IA bulging into LV LGO	4 nodules (T9 to L1)	T2 to L5	3 lesions including 1 nodule (LVs)	Yes
**B1** 5.9-year FS Boxer	IA—periventricular (LV) HGO		Midbrain to thoracic spinal cord		No
**B2** 5.6-year FS Fr Bulldog	IA bulging into LV HGO	2 nodules (cerebral)	Midbrain to thoracic spinal cord		Yes
**B3** 8.8-year FS Boxer	IA—periventricular (LV and V3) GBM		Surrounding midbrain	Periventricular hyperintensities (LV, V3, aqueduct)	Yes

*There were no cases with type III satellite tumors. Histological diagnoses were low grade (LG) or high grade (HG) of oligodendroglioma (O), astrocytoma (A), or glioblastoma (GBM)*.

*IA, intra-axial; LV, lateral ventricle; SAS, subarachnoid space; V3, third ventricle; V4, fourth ventricle*.

#### Group A

All seven cases initially presented with a focal neurolocalization based upon a neurological examination, had a solitary mass on the first MRI, and received treatment. In six cases, unilateral forebrain neurological deficits and seizures accompanied a singular prosencephalic mass on MRI. The other case presented for bilateral blindness with an optic chiasm–parasellar singular mass on MRI. After metastasis, additional neurological deficits included central vestibular syndrome (Cases A1 and A2), status epilepticus, bilateral forebrain deficits, worsening unilateral forebrain deficits, neck pain, or paraparesis (in Cases A3-A7, respectively).

**Figure 1:**
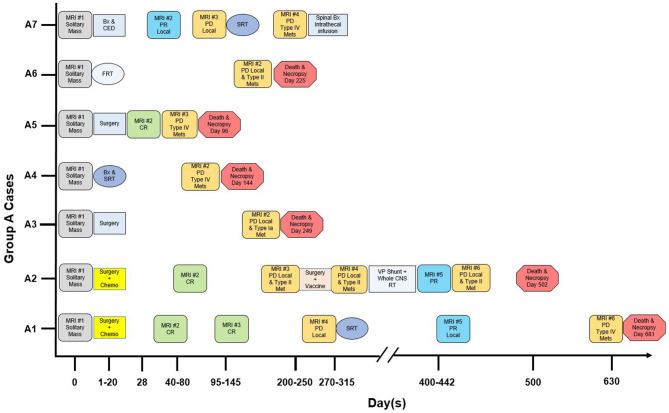
Magnetic resonance imaging (MRI), therapy and outcome in “Group A” dogs with CSF drop metastasis of glioma. Treatment response was designated as complete remission (CR), partial remission (PR), or progressive disease (PD) (9, 10). Bx, stereotactic biopsy. CED, convection-enhanced delivery of QUAD-doxorubicin (11). Chemo, CCNU and chlorambucil chemotherapy (12). FRT, fractionated radiation therapy (50 Gy total, 20 fractions). Intrathecal infusion, intrathecal infusion of QUAD-doxorubicin. Met, metastasis. SRT, stereotactic radiation therapy (15-24 Gy total, 1–3 fractions). VP, ventriculoperitoneal. Whole CNS RT, whole central nervous system radiation therapy (30 Gy total, 10 fractions).

The time course of diagnosis, therapies, and death in Group A is summarized in Figure 1. Therapy was directed locally at the mass, including excisional surgery (*n* = 4), stereotactic biopsy and convection-enhanced delivery (*n* = 1), stereotactic biopsy and stereotactic radiation therapy (SRT; *n* = 1), or fractionated radiation therapy (*n* = 1). All cases additionally received symptomatic therapy (glucocorticoids, anticonvulsant drugs), and two received enteral chemotherapy. In three dogs, progressive disease was treated [SRT for local recurrence in Cases A1 and A7; surgery then whole central nervous system (CNS) radiation for local recurrence and CSF drop metastasis in Case A2; intrathecal infusion of QUAD-doxorubicin for intraspinal drop metastases in Case A7].

Necropsy (six cases) confirmed the antemortem diagnosis of CSF drop metastasis, with (four cases) or without (two cases) recurrence of the primary mass. Case A7 was confirmed based on antemortem biopsies of the primary parietal glioma and, 7 months later, the spinal metastases.

#### Group B

This was three dogs with multifocal lesions on the original MRI. Case B1 had seizures (normal interictal neurological examination) and was euthanized the day after MRI. Case B2 (unilateral forebrain and bilateral C1–C5 deficits) had multifocal MRI lesions. Symptomatic treatment was administered (prednisolone, anticonvulsant drugs). On day 106, a second MRI revealed additional lesions. On day 135, the dog was euthanized. Case B3 (unilateral forebrain deficits, seizures) had a single MRI. Treatment was with uPA-targeted oncolytic Newcastle virus (three intravenous injections). Central vestibular deficits developed, and on day 17, the dog was euthanized. In all three dogs, complete necropsy revealed intracranial and spinal glioma.

**Figure 2:**
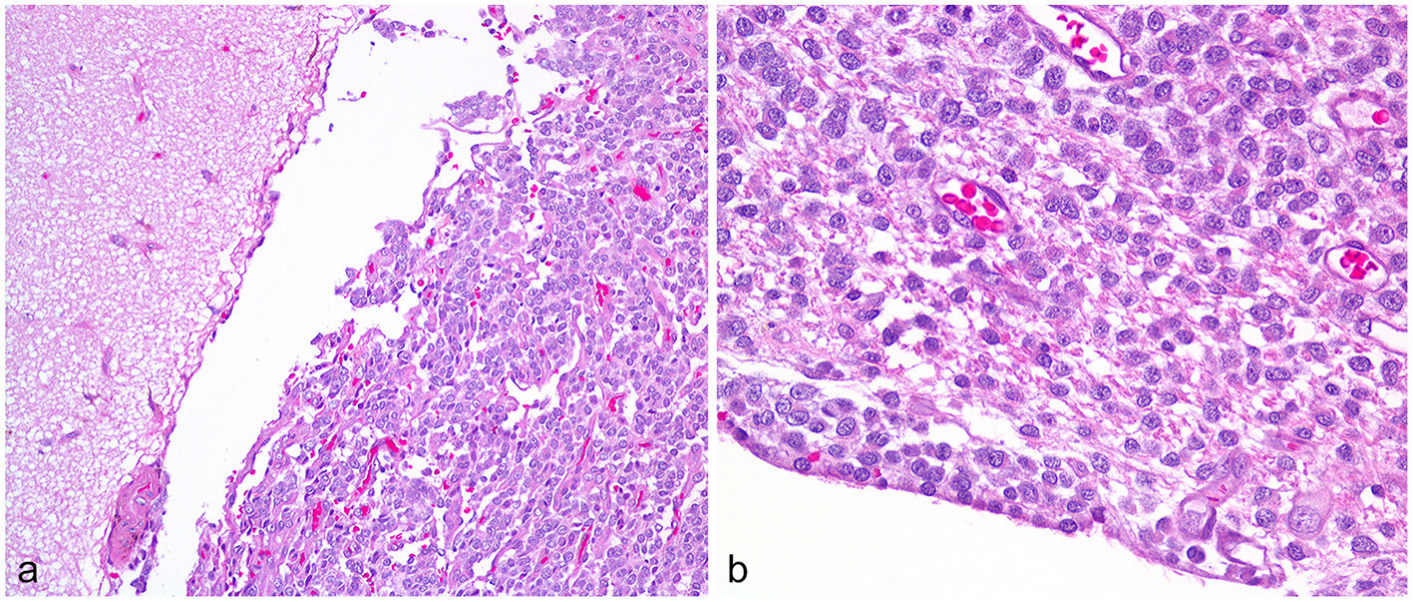
High-grade oligodendroglioma metastases, Case A2. **(a)** Cerebral leptomeningeal dissemination by the neoplastic cells involves the gyrus on the right, sparing the adjacent gyrus. Obj. 20x. **(b)** In a subependymal metastasis, the neoplastic cells abut the ependyma and contact the lateral ventricle at lower left. Obj. 40x.

In both groups, all 10 cases were histologically diagnosed with at least two anatomically distinct gliomas based upon antemortem biopsy/biopsies (six dogs) and necropsy (nine dogs). Immunohistochemistry included glial fibrillary acidic protein (five cases) and Olig2 (six cases). The final diagnosis was seven oligodendrogliomas (high grade *n* = 5; low grade *n* = 1; grade unknown *n* =1) and three astrocytomas (high-grade *n* = 2; glioblastoma *n* = 1). Metastatic lesions varied histologically (Figure 2), with individual cases having leptomeningeal, intraventricular, or periventricular subependymal lesions associated with regions such as the lateral, third, or fourth ventricles, mesencephalic aqueduct, optic nerves, and tracts, midbrain, cerebellum, subarachnoid space (SAS) of the skull base, spinal central canal, or leptomeninges of the cervical, thoracic, or lumbar spinal cord. Metastases matched the location of the MRI lesions discussed later, except that neoplastic invasion between cerebellar folia was also present histologically in Case B3.

### Magnetic Resonance Imaging

A total of 28 MRIs were evaluated. In Group A, 24 MRIs were assessed. Six MRIs, revealing a solitary mass at first presentation, were available for review. After surgery, there were a total of nine MRIs without any new lesions (four complete remissions, three partial remissions, and two recurrences of the primary mass only; Cases A1, A2, A5, and A7). Finally, in all seven dogs, new, distant lesions were revealed by nine MRIs.

All four MRIs performed on the dogs in Group B were reviewed.

Multiplanar sequences performed in all cases included T2-weighted (T2W), T2W-fluid attenuation fluid recovery (FLAIR), and T1-weighted (T1W; pre- and post-contrast). T2^*^-weighted gradient echo (GRE; nine cases) and diffusion-weighted imaging (eight cases) were also performed. Magnetic field strength was 1.5 T, except Case A3 (0.2 T).

### MRI Appearance: Primary Mass

**Figure 3:**
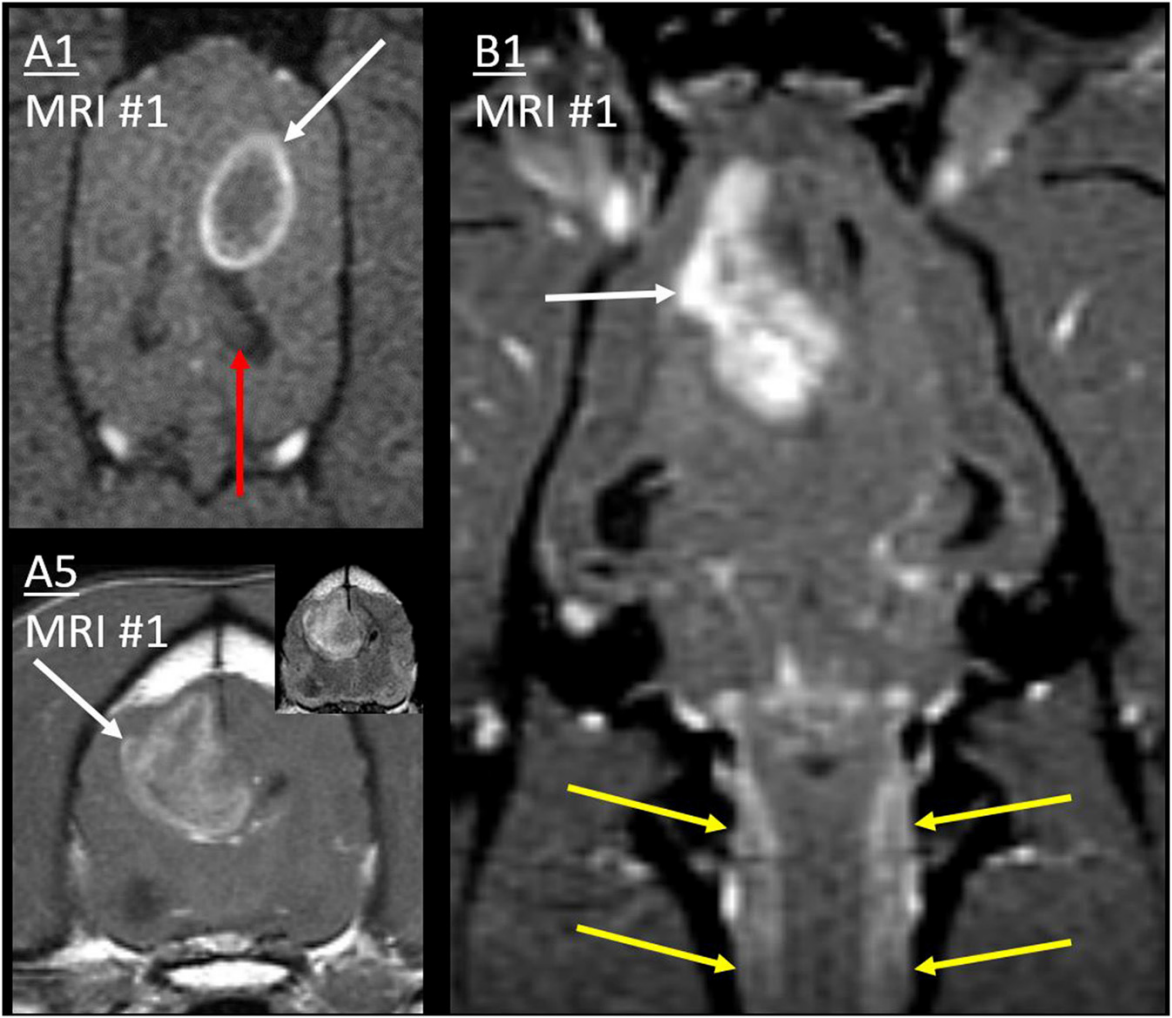
Primary masses. Post-contrast T1W images of cases A1 and B1 (dorsal with fat saturation) and A5 (transverse). Primary masses (white arrows) showed strong, peripheral, or heterogeneous contrast enhancement. Inset: Mass in A5 was heterogeneous on T2W-FLAIR. In all three cases, the border of the primary mass made intimate contact with the lateral ventricle, resulting in ventricular displacement. In B1, thick cervical leptomeningeal enhancement (yellow arrows) results from type 1b diffuse leptomeningeal CSF drop metastasis. Normal ipsilateral hippocampus in A1 (red arrow) would be affected by metastasis much later (Figure 9).

In all 10 cases, the primary mass contacted the CSF and was the largest and most rostral lesion. The association between the primary mass and the CSF can be seen in Figure 3 and other figures. Nine masses were intra-axial and in contact with a lateral ventricle, one of which also contacted the third ventricle. One mass was within the CSF of the optic chiasm and parasellar SAS.

As in Table 2, primary masses were generally heterogeneous on T2 and FLAIR, and heterogeneously contrast-enhancing. Although some regions of some tumors were strongly diffusion-weighted imaging—hyperintense, none of these regions were also hypointense on apparent diffusion coefficient mapping.

**Table 2:** Magnetic resonance imaging characteristics of the primary mass, in 10 dogs that developed cerebrospinal fluid (CSF) drop metastasis of glioma.

**MRI criteria**	**Number of cases**	
**T1 and T2 signals**	Heterogeneous (*n* = 7) Foci T2-isointense to CSF (*n* = 7)	Homogeneous T1 and T2 (*n* = 2) Homogeneous T1 only (*n* = 3)
**FLAIR signals**	Hetero with extreme hyperintense (*n* = 5) Hetero hyperintense (*n* = 1)	Half isointense, half extreme hypointense (*n* = 1) Homogeneous (*n* = 2)
**Gradient Echo signal voids (9 cases)**	Majority of mass (*n* = 1) Foci (*n* = 2)	None (*n* = 6)
**Perilesional edema** (adjacent T2/FLAIR hyperintensity)	Pronounced (*n* = 1) Negligible (*n* = 4)	Immediately adjacent only (*n* = 5)
**Contrast enhancement**	Strong peripheral (*n* = 4) Strong heterogeneous (*n* = 3)	Small region within non-enhancing mass (*n* = 2) Strong homogeneous (*n* = 1)
**Diffusion-Weighted Imaging (8 cases)**	Heterogeneous without extreme hyperintense *(n* = 6)	Heterogeneous with extreme hyperintense (*n* = 2)

*Extreme hyperintense: equal to signal of normal CSF on T2. Extreme hypointense: equal to signal of normal CSF on FLAIR*.

*FLAIR, fluid attenuation inversion recovery; Hetero, heterogeneous*.

Cases A4 and B2 had repeat MRI without surgical excision. In both cases, there was little change in the appearance of the primary mass. However, although the chiasmal mass itself was similar in Case A4, it had disseminated into adjacent leptomeninges (discussed later).

### MRI Appearance: Cerebrospinal Fluid Drop Metastasis Lesions

The metastatic lesions were classified according to the human system proposed by Bordignon et al. (5):

- Type I: Leptomeningeal dissemination
- Type Ia: nodular (presence of mass/nodule along the CSF cisterns)
- Type Ib: diffuse (diffuse leptomeningeal enhancement)
- Type II: Subependymal dissemination, e.g., ependymal enhancement
- Type III: Satellite tumor: the presence of nodular tumor distant from the primary lesion
- Type IV: Mixed: a combination of the above

All metastatic lesions were in contact with CSF in leptomeningeal or ependymal locations: there were no type III satellite tumors. Two dogs had type I lesions only, two dogs had type II lesions only, and six dogs had a type IV mixed combination of lesion types (Table 1). All abnormal signal areas on MRI shown later were histopathologically confirmed as metastatic lesions unless otherwise noted.

#### Type Ia: Nodular Leptomeningeal Lesions

**Figure 4:**
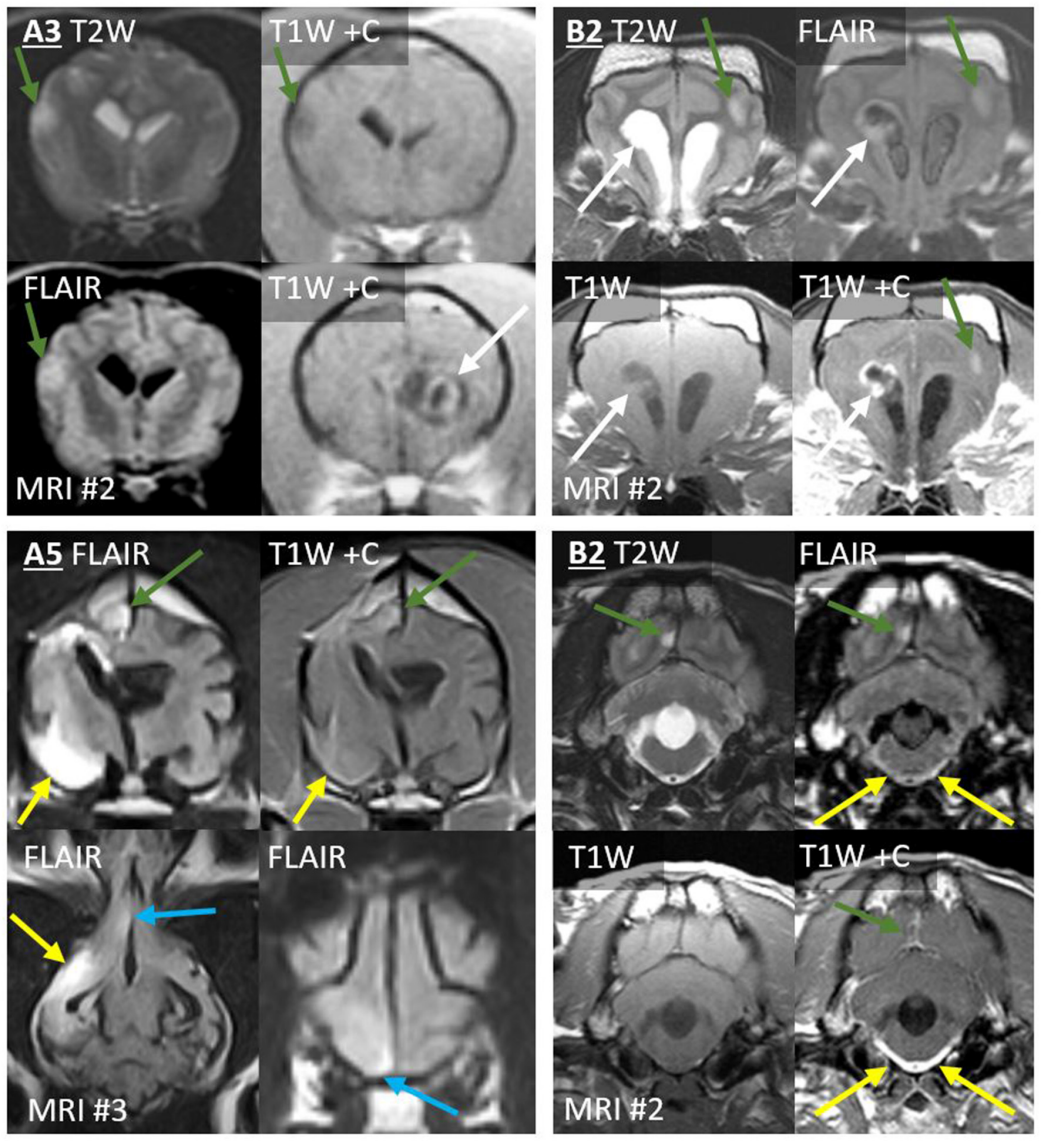
Type 1a CSF drop metastases: Leptomeningeal nodular lesions. Images of Case A3 (*top left*) are transverse (level of the internal capsule, except one image at the level of the primary mass). Images of Case A5 (*bottom left*) are 6 months post-operative (top, transverse images of the third ventricle; bottom, dorsal image of the ventral falx cerebri and transverse image of the rostral frontal lobe). Images of Case B2 are transverse at the level of the frontal lobes (*top right*) or at the level of the fourth ventricle (*bottom right*). Primary masses displayed heterogeneous signals and irregular contrast enhancement (white arrows). Leptomeningeal metastatic nodules (green arrows) did not resemble the primary mass, having homogenous signals, and being non-enhancing (A3), homogenously-enhancing (A5 and upper B2), or displaying only leptomeningeal enhancement (lower B2). In Case A5, largest metastasis (yellow arrows) displayed extensive parenchymal FLAIR-hyperintensity with leptomeningeal enhancement. Additionally, ependymal FLAIR-hyperintensity and contrast enhancement extend from the resection cavity. A leptomeningeal metastatic nodule (blue arrows) in the ventral frontal lobe abuts the falcine CSF. These lesions had not been present on the pre- or post-operative MRIs. In Case B2, the three leptomeningeal nodules (green arrows) had not been present on MRI #1. T2-hyperintense and T1-hypointense signals extend ventrally from the rostral horns. There is concurrent, very thick, and strong leptomeningeal enhancement ventral to the medulla and surrounding the basilar artery; this lesion is FLAIR-hyperintense (yellow arrows). Fourth ventricle is severely enlarged.

In Case A3, the second MRI revealed a single new T2-hyperintense nodule in the contralateral temporal cortex, merging into the SAS on T2W (Figure 4). On both MRIs, the primary mass was homogeneously extremely T1-hypointense and T2-hyperintense (CSF-isointense), with a region of strong contrast-enhancement. The new lesion was less T2-hyperintense, much less T1-hypointense, and non-enhancing. The drop metastasis was also different on T2W-FLAIR, being homogeneously hyperintense to gray matter (GM), whereas the primary mass was half extremely hypointense (nearly CSF-isointense) and half GM-isointense.

The third MRI performed on Case A5 revealed five leptomeningeal nodules, none of which had been observed on earlier MRIs (Figure 4): four ipsilateral to the primary mass (frontal lobe, pyriform lobe, cingulate gyrus, and parahippocampal gyrus) and a very small contralateral parahippocampal lesion. Each was a moderate-strong T2-hyperintensity of the GM immediately subjacent to the SAS, continuous with the CSF on T2W images. All were strongly hyperintense and conspicuous on T2W-FLAIR. T2 and FLAIR signals were homogenous or mildly heterogeneous. These lesions were not visible on T1 (GM-isointense). In contrast, on the preoperative MRI, the primary mass had been very heterogeneous on T2 and FLAIR (Figure 3) and highly evident on T1 (GM-hypointense). Enhancement of the metastases varied: the frontal lobe metastasis did not, whereas there was a homogeneous parenchymal enhancement of the cingulate and parahippocampal lesions. The temporal lesion showed strong leptomeningeal enhancement. Again, none of the metastases matched the strong, irregular intra-axial enhancement of the primary mass (Figure 3).

**Figure 5:**
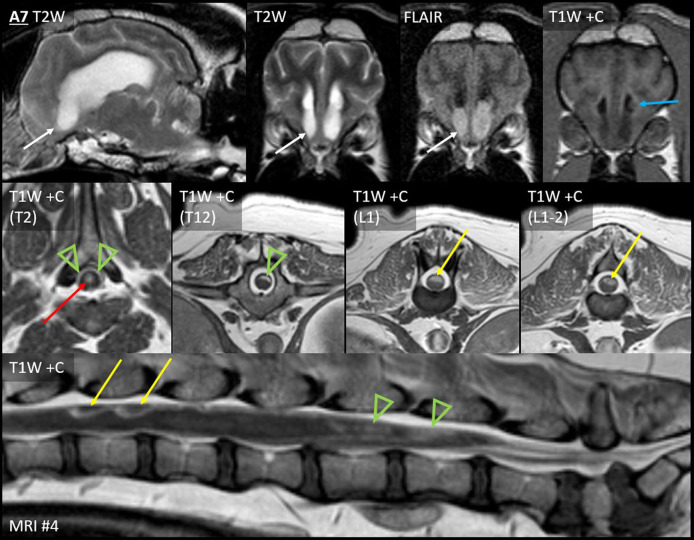
Leptomeningeal dissemination (nodular type Ia and diffuse type Ib) and subependymal dissemination (type II), Case A7. Seven months after CED for a parietal lobe mass, Case A7 developed paraparesis. *Top row:* Parasagittal T2W and transverse T2W, T2W-FLAIR and T1W post-contrast images of the rostral horns. *Middle row:* T1W post-contrast transverse images at the level of the T2, T12, L1, and L1–L2 vertebrae. *Bottom row:* T1W post-contrast sagittal image of the lumbosacral vertebrae. Abnormal material fills the ventral aspect of both rostral horns (white arrows). Material is nearly T2-isointense to adjacent CSF but is distinctly hyperintense to CSF on FLAIR. The material is non-contrast-enhancing; an enhancing ependymal nodule is located just dorsally within the left ventricle (blue arrow). This accompanied irregular thickening and enhancement (green arrowheads) of the dorsal leptomeninges from T1 to L4, the conus medullaris, and the accompanying nerve roots. There were also four distinct nodules: two are shown (L1 and L1–L2, yellow arrows). Central canal ependymal enhancement was present at T2–T4 (red arrow).

On the fourth MRI, Case A7 developed four discrete leptomeningeal nodules, with accompanying diffuse leptomeningeal enhancement (discussed later). These nodules were dorsal to the spinal cord at T9, T12, L1, and L1–L2 (Figure 5).

The first MRI in Case B2 had shown diffuse leptomeningeal enhancement (discussed later) in addition to the primary mass. After symptomatic therapy, two nodular metastases were present on the second MRI (Figure 4). Ipsilateral to the primary mass, the occipital lesion made broad-based contact with the overlying SAS. The contralateral frontal lesion surrounded a sulcus and was therefore also contiguous with the CSF. Both occurred as homogeneous T2W and T2W-FLAIR hyperintensity of cortical GM. There was leptomeningeal enhancement overlying the occipital lesion and moderate, homogeneous enhancement of the frontal lesion. These lesions were not evident pre-contrast (T1W GM-isointense). In contrast, the primary lesion had heterogeneous signals, including extreme T1-hypointensity and T2-hyperintensity, with strong, peripheral contrast enhancement.

#### Type Ib: Diffuse Leptomeningeal Enhancement

In Cases A1, B1, and B2, there was uninterrupted, strong, thick leptomeningeal contrast enhancement that fully encircled the cervical spinal cord, beginning at the caudal medulla oblongata. It was distant from, and entirely dissimilar to, the intra-axial primary cerebral mass of each case.

**Figure 6:**
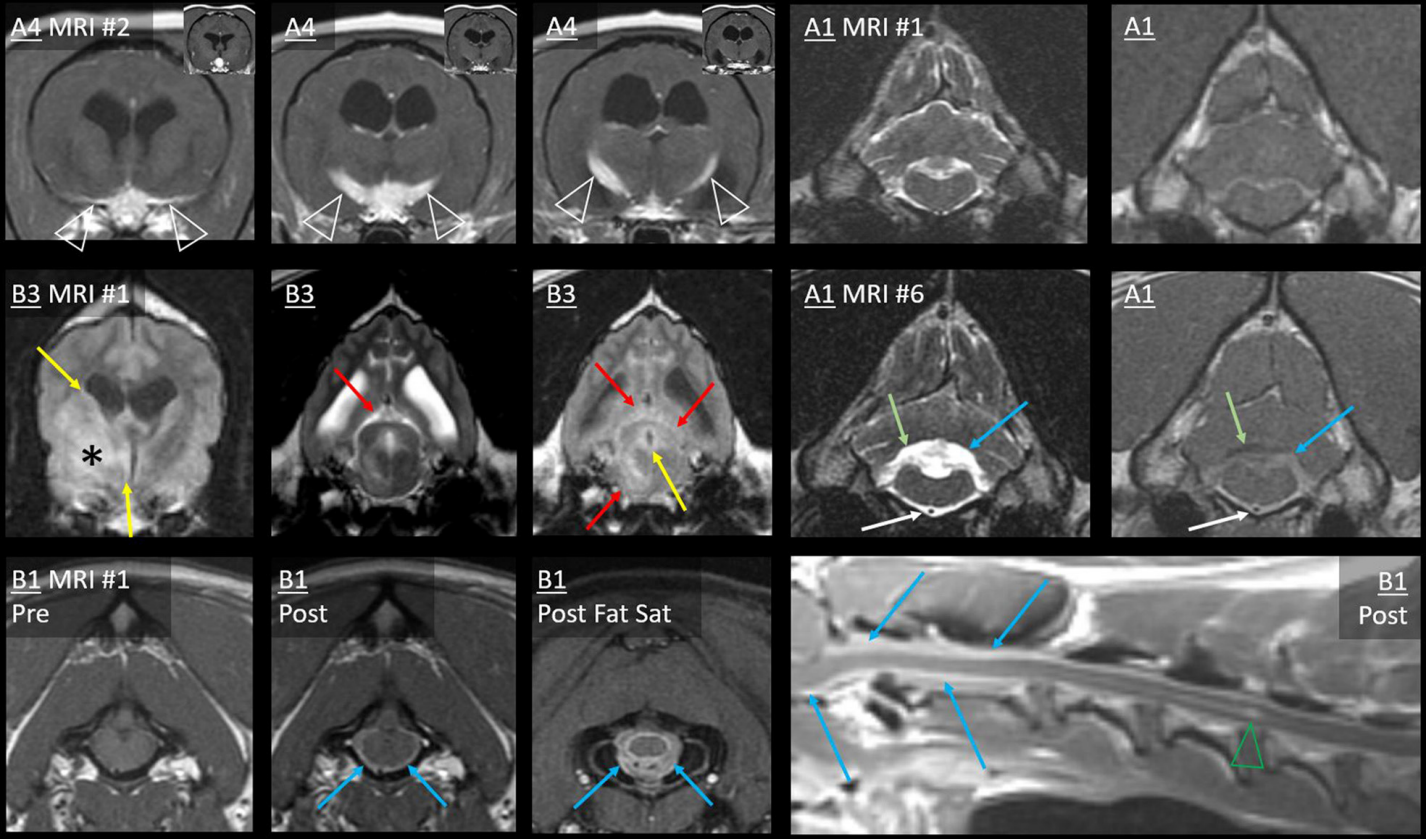
Type 1b dissemination: Diffuse leptomeningeal lesions. Case A4: post-radiation transverse T1W post-contrast images (level of the optic chiasm and tracts). A contrast-enhancing mass effaces the chiasma. Thin, strong leptomeningeal contrast enhancement extends over both ventral cortical surfaces (arrowheads, first image). Very thick leptomeningeal enhancement follows the optic tracts dorso-caudo-laterally (arrowheads, second and third images). Bilateral ventriculomegaly is present. Inset: pre-radiation images—the primary mass is present without any leptomeningeal lesion. Case A1: transverse T2W and T1W post-contrast images from before (MRI #1) and after surgery (MRI #6) for a parietal mass. On MRI #6, thick and strong leptomeningeal contrast enhancement and T2-hyperintensity have developed ventral to the medulla (white arrows), encasing the basilar artery. Additionally, the height of the T2-hyperintense fourth ventricle and contrast-enhancing choroid plexus has considerably increased (blue arrows). There are T2-hyperintensity and T1-hypointensity in the cerebellum dorsal to the enhancement (green arrows). In Case B3, transverse FLAIR (level of the third ventricle) and T2W and FLAIR (level of caudal aqueduct) images show the primary mass (asterisk). Periventricular FLAIR-hyperintensity (yellow arrows) affects the lateral and third ventricles and the periaqueductal GM (PAG). On the T2W image, there is poor distinction between aqueductal CSF and PAG. Midbrain SAS has a normal appearance on T2W, but CSF is markedly FLAIR-hyperintense (red arrows). Neoplastic cells were present in subependymal (ventricles and PAG) and subarachnoid (including midbrain) sites at necropsy. T1-weighted images of Case B1: transverse at the level of the foramen magnum (pre- and post-contrast), the atlanto-occipital joint (post-contrast with fat saturation), and post-contrast mid-sagittal cervical. There is diffuse cervical leptomeningeal enhancement extending from caudal medulla oblongata (blue arrows). On fat saturation image, thick leptomeningeal enhancement surrounds bifurcating ventral spinal artery and spinal cord. Central canal ependymal enhancement crescendos at C4–C5 (green arrowhead).

This leptomeningeal enhancement extended ventrally to the level of the midbrain. In Case A1 (Figure 6), it extended to the caudal boundary of the MRI (level of C1), and there was pronounced ventriculomegaly (all four ventricles, with interstitial edema of rostral horns and other periventricular locations). The other two cases also had spinal MRI. In Case B1 (Figures 3, 6), the encircling leptomeningeal contrast enhancement was continuous from the medulla to the second thoracic vertebra. There was also ventriculomegaly (lateral ventricles), syringomyelia (C2–3), and central canal ependymal enhancement (C4–5).

**Figure 7:**
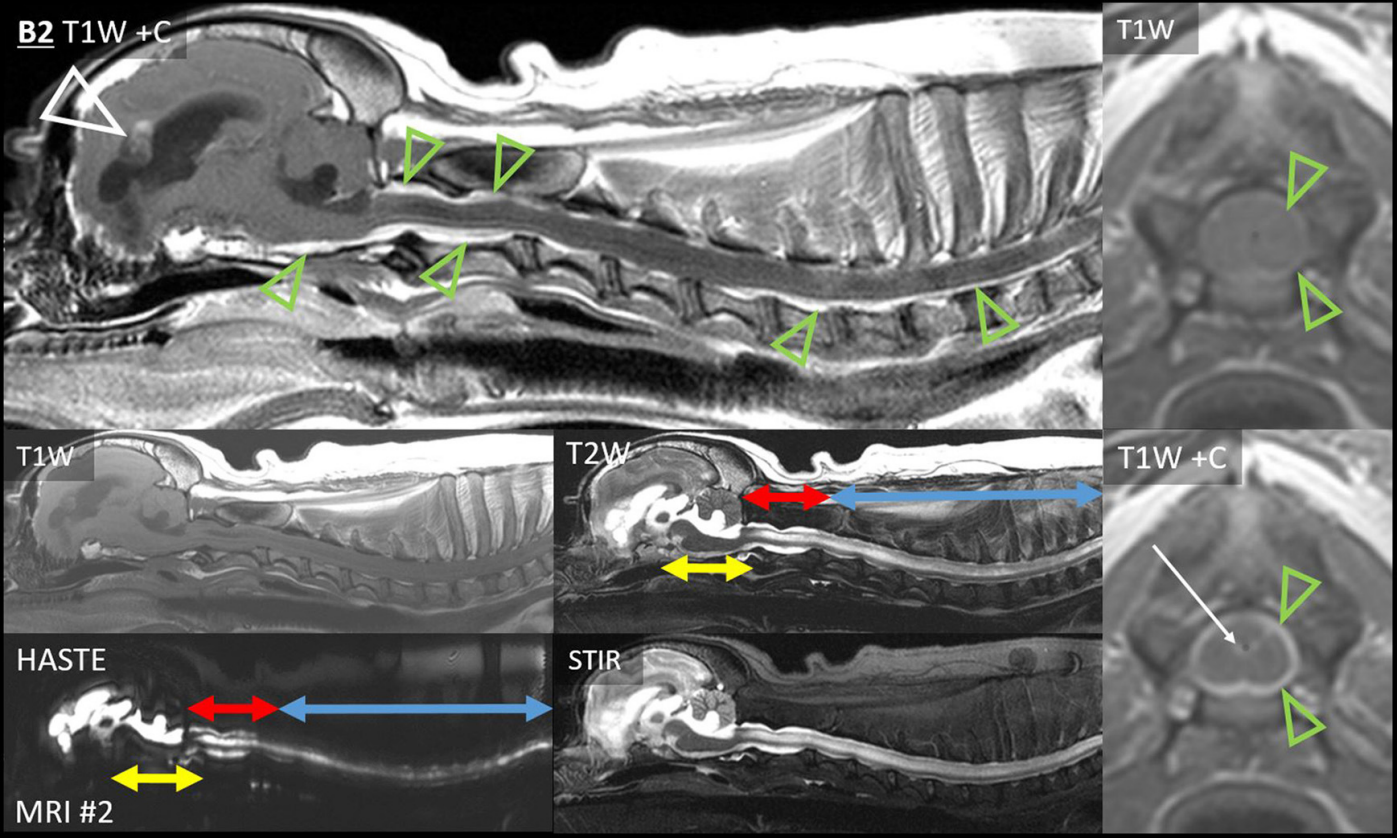
Extensive leptomeningeal dissemination and generalized hydrocephalus, Case B2. Sagittal images of the brain and cervicothoracic spinal cord: T1W pre- and post-contrast, T2W, HASTE, and T2W-STIR. Normal CSF cannot be visualized ventral to the brainstem or in the cervical SAS. Note the lack of normal SAS on HASTE combined with the lack of normal epidural fat on T1W pre-contrast. Yellow double-headed arrows: CSF ventral to brainstem is irregular and hypointense to normal CSF on T2, STIR, and HASTE. Red double-headed arrows: within C1–C2, an irregular SAS can be identified on HASTE but not on T1, and it is strongly contrast-enhancing. Blue double-headed arrows: cervicothoracic SAS is difficult to identify on HASTE and contrast-enhancing. Narrow T2-hyperintensity dorsal and ventral to the spinal cord does not suppress on STIR and is not evident on T1W pre-contrast, suggesting it is composed of CSF with attenuation of epidural fat. There is widespread thick, strong leptomeningeal contrast enhancement (green arrowheads), including ventral to the brainstem and C1–C2, and dorsally from the obex to C1–C2. Caudal to C2, strong leptomeningeal enhancement was particularly evident on transverse images. T1W pre- and post-contrast transverse images, level of C3: normal epidural fat is barely discernible. There is thick, strong leptomeningeal contrast enhancement (green arrowheads), ependymal enhancement (white arrow), and intramedullary mass effect. There is severe ventriculomegaly of all ventricles, with partial volume averaging of the rostral horn lesions and the falx cerebri. Irregularly contrast-enhancing primary intra-axial mass bulges into the lateral ventricle (white arrowhead).

The leptomeningeal thickening in Case B2 (Figures 4, 7) was exceedingly thick and strong. Abnormal CSF signals were beginning ventral to the brainstem and encircling the spinal cord to the boundary of the scan (T6). As in Figure 7, a combination of intramedullary mass effect and leptomeningeal thickening displaced the cervical epidural fat almost to the point that it could no longer be identified. Although T2-hyperintense material could be seen surrounding the spinal cord initially suggesting epidural fat, closer comparison with short tau inversion recovery (STIR), T1 pre- and post-contrast, and half-Fourier acquisition single-shot turbo spin-echo (HASTE) images indicated that this T2-hyperintensity was actually infiltrated SAS. This abnormal and contrast-enhancing SAS continued ventrally to the brainstem, where there is no epidural fat. There was pronounced ventriculomegaly (all ventricles), with severe interstitial edema around the rostral horns.

Additionally, there was irregular intramedullary T2, STIR, and HASTE hyperintensity, consistent with syringomyelia or spinal cord edema. It was mainly T1-isointense to GM, but there was also a dilated central canal with no signal on T1W or FLAIR (C1–C2), ependymal enhancement (C3), and T1-hypointense GM (C5–C7). A thoracic T2-sagittal sequence documented further dorsal intramedullary hyperintensity (T4–T12).

In case A7 (Figure 5), the spinal leptomeningeal nodules (discussed earlier) were accompanied by enhancement of the dorsal leptomeninges (irregularly from T1 to L4), the conus medullaris (L4–L5 vertebrae), and the ependyma (central canal within T2–T4).

Case A4 presented a different type of leptomeningeal enhancement. On the second MRI, leptomeningeal contrast enhancement extended directly from the previously diagnosed chiasmal mass (Figure 6). Strong and very thick leptomeningeal enhancement followed both optic tracts dorso-caudo-laterally. Strong leptomeningeal enhancement also extended laterally (ventral to both pyriform lobes); this was thinner than the optic tract lesions. Contrast enhancement extended into the ventral aspect of the third ventricle. There had been no leptomeningeal enhancement on the earlier MRI.

Finally, the SAS surrounding the midbrain of Case B3 contained FLAIR-hyperintense CSF (Figure 6). There was no contrast enhancement.

#### Type II: Subependymal Dissemination

**Figure 8:**
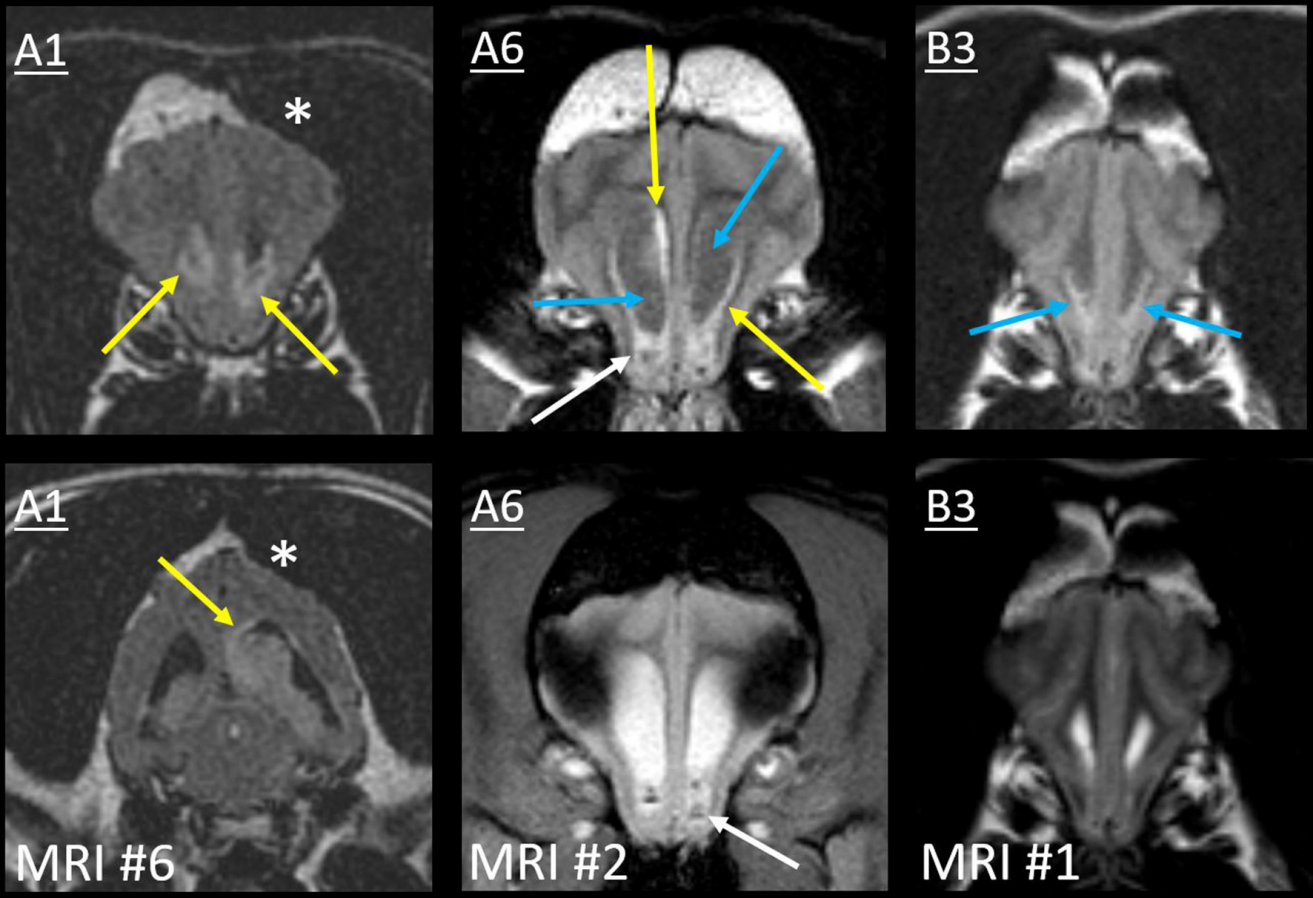
Type II ependymal CSF drop metastasis. Images from Cases A1, A6, and B3 (*left, middle*, and *right)*. Transverse MRIs at the level of the rostral horn (lower image of A1: level of the tail of the hippocampus). Images are T2W-FLAIR, except the lower images of A6 (T2*W GRE) and B3 (T2W). A surgical bone defect is evident (A1, asterisks). On FLAIR, ependymal hyperintensity incompletely surrounds the lateral ventricles (yellow arrows). Note the symmetrical failure of CSF suppression on FLAIR (blue arrows). In Case A1, there is a hippocampal nodule. Abnormal material pools bilaterally in the rostral horns of Case A6 (white arrows): it is heterogeneous on FLAIR with GRE signal voids. There are symmetrical magnetic susceptibility artifacts lateral to both ventricles due to the air-tissue interface of the frontal sinuses. In Case B3, T2W appearance of the rostral horns is unremarkable, yet no normal CSF is present on FLAIR (blue arrows).

The sixth MRI of Case A1 revealed five new ventricular lesions. Ipsilateral to the previously excised mass, there was a heterogeneously T2-hyperintense hippocampal mass bulging into the lateral ventricle, with a single line of mild ependymal contrast enhancement (Figure 8). Next were paired lesions where abnormal signals replaced the lumen of both rostral horns. They were highly evident on T2W-FLAIR, being heterogeneous and strongly hyperintense to CSF (GM-hyperintense). These non-enhancing lesions were otherwise almost inconspicuous: compared with normal CSF, the lumina were only slightly T2-hypointense and T1-hyperintense. An even smaller lesion was T2-heterogeneity within the ventral third ventricle, with FLAIR peri-ventricular hyperintensity. Finally, the fourth ventricle and the choroid plexus were considerably taller than on all five prior MRIs (Figure 6). The primary tumor had been intra-axial and strongly peripherally enhancing (Figure 3). Pronounced adjacent white matter tract edema had affected almost half the ipsilateral hemisphere. In contrast, these new lesions were intraventricular or ependymal, non-enhancing except the single hippocampal line and displayed no perilesional edema.

**Figure 9:**
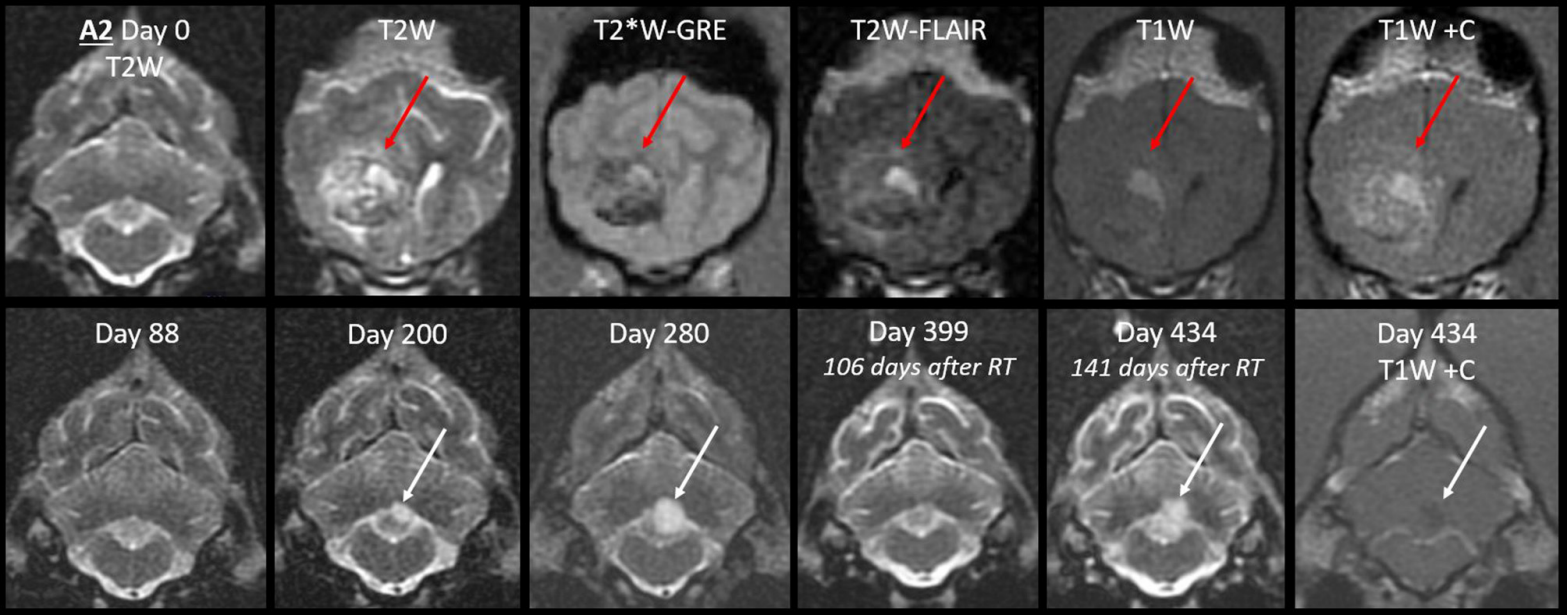
Progression of the cerebellar lingula CSF drop metastasis, Case A2. *Top row:* the normal cerebellum and the primary mass before surgery (T2W, T2*W-GRE, T2W-FLAIR, and T1W pre- and post-contrast). The primary mass (red arrows) effaces the lateral ventricle and is tremendously heterogeneous, hemorrhagic, irregularly contrast-enhancing, and space-occupying. *Bottom row:* cerebellar lesion over time, transverse T2W, and T1W post-contrast images. Homogenous T2-hyperintense lingula lesion was first detected 6 months after surgery (white arrows). It increased in size, then resolved with whole CNS radiation therapy. On day 434, it reached its largest size and became mildly T2-heterogeneous. It was non-enhancing and mildly T1-hypointense. At no stage did it resemble the primary mass.

In Case A2, the cerebellar lingula became asymmetrically T2-hyperintense on MRI #3 (Figure 9). This region was normal on both prior MRIs. The new lesion was small, homogeneous (T2W and T2W-FLAIR), not evident on T1 (GM-isointense), and non-enhancing. It was very different from the primary tumor, which was composed of coalescing GRE signal voids, with extreme heterogeneity on T1, T2, and FLAIR (from no signal to extreme hyperintensity) and irregular contrast enhancement. On repeat MRI 80 days later, the lingula lesion was larger but still homogenously T2-hyperintense. It was now visible on T1W images as a homogeneous hypointensity. After whole CNS irradiation, the cerebellum returned to normal appearance. On the sixth MRI, 5 months after radiation, the lingula lesion was now the largest ever, with some T2-heterogeneity for the first time. There was still no contrast enhancement. This MRI also revealed a small, strongly T2-hyperintense, mildly T1-hypointense, non-contrast-enhancing nodule immediately ventral to one rostral horn.

The first MRI of Case A4 had shown periventricular FLAIR-hyperintensity (lateral and third ventricles). On the next MRI after radiation therapy, these regions merged into largely symmetrical white matter T2-hyperintensity, and there was progressive lateral ventricle ventriculomegaly.

In Case A5 (many type Ia nodules, discussed previously) on the third MRI, there was a new linear FLAIR-hyperintensity and contrast enhancement in the ependyma (Figure 4). It was continuous with the surgical resection cavity, but it had not been present on the immediately post-operative MRI.

**Figure 10:**
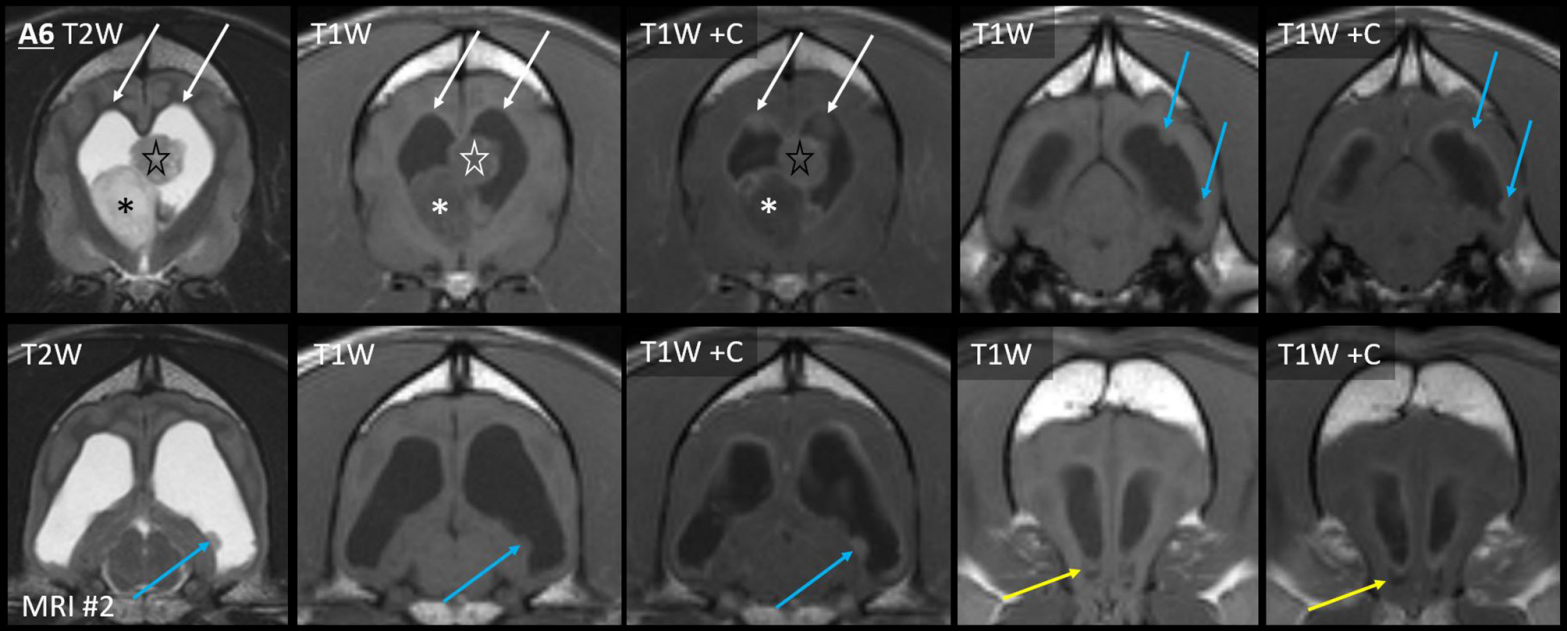
Myriad type II ependymal lesions, Case A6. Transverse images from MRI #2 (level of the interventricular foramina, which have been effaced; level of the occipital lobes; level of the aqueduct; and the level of the rostral horns). The previously diagnosed primary mass is evident (asterisk). There are now metastatic ependymal nodules in both lateral ventricles, including the septum pellucidum (star) and five others (white and blue arrows). On T2W and T1W pre-contrast images, the dorsal parietal nodules are not visible (white arrows), whereas the other ependymal nodules are evident (blue arrows). There is also widespread, irregular ependymal contrast-enhancement (all post-contrast images). Yellow arrows highlight ventral rostral horn subependymal lesions.

On the second MRI of Case A6, the ependyma of both lateral ventricles was irregularly contrast-enhancing, thickened, and FLAIR-hyperintense (Figures 8, 10). The CSF failed to fully suppress on FLAIR. This high-protein CSF was present in the lateral ventricles only; CSF elsewhere had a normal suppressed FLAIR signal. The ventral rostral horns contained heterogeneous, non-enhancing material and GRE signal voids, suggesting dependent pooling of intraventricular hemorrhage.

Arising within this thickened ependyma were at least six ependymal nodules (septum pellucidum, left parietal, right parietal, left temporal, and two left occipital) (Figure 10). Each protruded into the lumen of the lateral ventricle and was highly conspicuous post-contrast, being homogeneously moderately-strongly enhancing. The left parietal lesion formed a pedunculated mass. The parietal pair of nodules were imperceptible before contrast administration, being isointense to the surrounding high-protein CSF on each of T1, T2, and FLAIR. The other four nodules were visually distinct pre-contrast: compared with the surrounding CSF, they were T1-hyperintense, T2-hypointense, and FLAIR-hyperintense. Compared with GM, they were T1-isointense, T2-isointense, and FLAIR-hyperintense. The septum pellucidum nodule was the largest. It abutted the primary mass, was mildly T2 and FLAIR heterogeneous, and had pinpoint GRE signal voids. The other nodules were homogeneous on every sequence without GRE voids. In contrast to these metastatic nodules, the primary mass was intra-axial, T2-heterogeneous with foci isointense to CSF, had pinpoint GRE signal voids, and did not enhance. It bulged prominently into one lateral ventricle. The overlying ependyma enhanced strongly, producing a segment of peripheral enhancement. Obstructive hydrocephalus was seen as enlargement of both lateral ventricles, diffuse gyral flattening, attenuation of the third ventricle, and transtentorial herniation.

Concurrent to the development of thoracolumbar leptomeningeal lesions, Case A7 developed rostral horn lesions (Figure 5). The ventral aspect of both horns was filled by nearly homogeneous material that was strongly FLAIR-hyperintense to normal CSF (GM-hyperintense) and thus highly conspicuous. It was less evident on other sequences (compared with CSF, it was mildly T1-hyperintense and T2-hypointense, without GRE signal voids). There was no enhancement. Immediately dorsal, there was one moderately, homogenously-enhancing ependymal nodule. These ventricular lesions were very different from the intra-axial primary mass, which had been greatly heterogeneous and strongly irregularly enhancing. Although the thoracolumbar metastases were biopsied and confirmed to be metastatic, these ventricular lesions were not histologically assessed.

In Case B3, a single MRI revealed subtle signal changes accompanying the primary mass. There was ependymal FLAIR-hyperintensity where the primary mass contacted the lateral and third ventricles (Figure 6). Distant to this, homogeneously T2W and T2-FLAIR hyperintense parenchyma surrounded the aqueduct. This caused poor T2W distinction between the CSF and peri-aqueductal GM. The rostral horns were FLAIR-hyperintense (Figure 8). As discussed earlier, the SAS around the midbrain was also abnormal. The primary mass was intra-axial, heterogeneous on all sequences with a focus of contrast enhancement, and quite unlike these small non-enhancing metastatic lesions.

## Discussion

The following major findings will be discussed. Glioma CSF drop metastases may differ markedly from the primary mass on MRI, with nodules showing more homogeneous signals and less contrast enhancement or entirely different leptomeningeal enhancement. After diagnosing an intra-axial mass, leptomeningeal or ependymal lesions may arise, with combinations of lesion types (type IV) being common. Although metastases can be clinically devastating, some are comparatively subtle on imaging. Metastatic glioma lesions may become increasingly recognized after aggressive treatment of the primary tumor, but they are also seen at first presentation.

The metastatic lesions in this study were markedly dissimilar to the primary mass on MRI. The location was different, with intra-axial masses spreading to ependymal (intraventricular or periventricular) or leptomeningeal (extra-axial or superficial intra-axial) sites. Diffuse meningeal enhancement reminiscent of meningitis was a common finding. The nodular metastases displayed very different signals to the primary mass. Heterogeneous contrast-enhancing gliomas caused homogeneous non-enhancing nodules. Examples of metastases deviating from the primary mass in key imaging criteria include an enhancing primary mass with a non-enhancing leptomeningeal nodule (Figure 4), a primary mass with extreme heterogeneity on all sequences with a homogeneous ependymal nodule (Figure 9), and diffuse extra-axial (leptomeningeal) enhancement accompanying an intra-axial mass (Figure 7). There might be multiple reasons why metastatic lesions differed in appearance. For metastasis to the parenchyma, these relatively early, smaller lesions may have not yet developed necrosis, hemorrhage, and resulting heterogeneous signals. Diffuse metastasis to the leptomeninges naturally appeared as very different to the primary intra-axial mass on MRI. We confirmed the hypothesis that CSF drop metastases generally have different MRI characteristics to the primary mass.

Most patients had multiple metastatic lesions; some developed yet more metastases over time. Patient follow-up (up to 2 years and up to six MRIs per patient) was valuable in chronicling the evolving disease process. Each of type Ia, type Ib, and type II lesions were observed in multiple dogs. Type IV, a mixed combination of these lesion types, was overall most common.

These CSF drop metastases developed in varying periventricular and leptomeningeal locations without any strong predilections. However, with one exception, metastases did occur in accordance with the direction of CSF flow. Primary tumors were within or bulging into the lateral ventricle, and metastatic lesions were within the lateral ventricles, in more caudal components of the ventricular system, or in the subarachnoid space. Rostral horn lesions were either intraventricular (e.g., lack of CSF suppression on FLAIR or intraventricular GRE signal voids) or periventricular (e.g., ependymal enhancement or interstitial edema secondary to hydrocephalus). The exception was the optic chiasm mass with direct leptomeningeal extension in Case A4. This mass invaded into the third ventricle, with periventricular infiltrates in the third and lateral ventricles on MRI and necropsy. Obstructive hydrocephalus and reduced CSF flow may have allowed retrograde CSF drop metastasis. In 40% of our cases, cervical and even thoracolumbar spinal metastases were seen. It is imperative to note that in cases presenting for intracranial disease, spinal imaging was necessary to accurately document the extent of the neoplasm. Recent work has shown the substantial upward movement of CSF during forced inspiration (13). This study found that net CSF flow was actually upward during a predefined breathing protocol of normal and forced inspiration and expiration in 12 healthy supine human subjects. There could also a role for gravity in addition to the direction of CSF flow, which is thought to be the reason spinal CSF drop metastasis of human glioblastoma is most commonly to the lower thoracic, lumbosacral, cauda equina, and thecal sac regions (14). Similarly, in our quadruped patients, when the caudal fossa was affected, it was always the leptomeninges ventral to the brainstem (Cases A1, B1, and B2) and never the lateral or dorsal leptomeninges.

Another key finding is that a few of the metastatic lesions were subtle. T2W-FLAIR sequences proved useful, displaying easily apparent lesions when T1 and T2 signals were close to normal, and contrast-enhancement was lacking. The glioblastoma (Case B3) with unilateral forebrain deficits had the least obvious MRI findings of metastasis. In the rostral horns (Figure 8) and the SAS around the midbrain (Figure 6), there were subtle FLAIR lesions despite normal T2W appearance, and the cerebellar folia were normal. Just 17 days later, central vestibular deficits necessitated euthanasia, and all three locations were found to contain histological CSF drop metastases. In Case A2, the metastasis was small and homogenous at first but became larger and T2-heterogenous over time (Figure 9). Even after months of treatment and recurrence, this lesion remained smaller and much more homogeneous than the primary tumor and non-enhancing. Although CSF drop metastases varied from inconspicuous to obvious, they were clinically devastating; seven cases were euthanized within 1 month of the first detection. Similarly, in humans, most cases are deceased within months of dissemination, regardless of the type of dissemination (5). Although the type of metastasis is not yet associated with prognosis, it is valuable to recognize that glioma CSF drop metastases can take vastly different MRI appearances, from non-enhancing periventricular lesions to diffusely enhancing leptomeningeal lesions. The different MRI appearances may reflect different pathophysiology, such as dissemination of free-floating cells *via* the CSF vs. migration under the ependyma (5, 15, 16). Examples of both are seen in Figure 2, with a knot of glioma cells in the leptomeninges sparing the adjacent parenchyma (*left*) contrasting with dense infiltration of neoplastic cells under the ependyma and no neoplasm evident within the ventricle (*right*).

It is worth noting that in most treated dogs (Group A), therapy was very much restricted to the primary mass, such as excisional surgery, SRT, or CED. In glioma surgery, opening of the ventricle is common, contributing to the risk of CSF drop metastasis (5). Only two dogs were additionally administered enteral chemotherapy, Cases A1 and A2. The CCNU and chlorambucil protocol is described elsewhere (12). After completing the 5-month course of CCNU, these two cases had relatively delayed detection of metastasis (months 21 and 7 from the first MRI, respectively) and the longest overall survival (17 and 23 months, respectively). In the other Group A cases, metastasis was often detected earlier (3-7 months), and the overall survival was less (3-8 months, with one case still alive). It seems possible that the advent of surgery and radiation therapy may allow an increased development of delayed CSF drop metastasis. This is supported by our previously reported case with a primary cerebral tumor treated by SRT (6). The first CSF drop metastasis to the fourth ventricle was also treated locally with radiation. Diffuse leptomeningeal and ependymal (brainstem and cervical) metastases were present on the final MRI and on necropsy (6). The current cases also displayed evolution in neurolocalization, such as the development of central vestibular syndrome or paraparesis in cases with cerebral tumors or the development of bilateral forebrain deficits in a case of chiasmal blindness.

It was not surprising that most primary tumors were oligodendrogliomas, as these occur more commonly in association with ventricles than astrocytomas (1, 4). Primary tumors had similar MRI characteristics to previous reports of solitary gliomas (1–3), including distortion of ventricles which is typical in oligodendrogliomas (1). In humans, in which astrocytoma predominates, satellite lesions (which do not spread *via* CSF and might occur *via* white matter) are a common form of intra-cerebral dissemination (5). In dogs, metastasis *via* the CSF has been well-documented, with all previously reported cases and most in the current study being oligodendrogliomas (6, 7). The number of cases in this study is, however, too few to draw reliable conclusions about differences between canine oligodendroglioma and astrocytoma. The signalment was also to be expected, primarily middle-aged, brachycephalic breeds of dog. High tumor grade should predict malignant behavior, and most tumors (80%) in this study were high grade.

This study's limitations include that, although 28 MRIs were available for review, only 13 MRIs showed CSF drop metastases. Still, this is a significant advance on the limited number of MRIs performed on all previously published glioma CSF drop metastasis. Furthermore, the MRIs performed many months earlier on the same cases, showing only a primary mass or post-treatment remission, did help to support that these lesions are occurring *via* CSF drop metastasis. Some glial neoplasms might occur as a naturally multifocal disease process, with *de novo* multifocal MRI lesions. However, the multifocal lesions in these cases appeared to occur *via* sequential metastasis and close to CSF pathways. The Bordignon et al. report of human glioma dissemination seems to indicate all lesions due to subependymal dissemination displayed contrast enhancement (5). Although there was ependymal enhancement in our Cases A1 and A5–A7, there was no enhancement of the ependymal lesions in Cases A2, A4, and B3 and of other lesions in A1 and A7. This might reflect the higher proportion of primary gliomas that contrast-enhanced in humans rather than dogs. Other limitations include varying MRI protocols, machines, and institutions. In one case, imaging was performed on a low-field magnet, but image quality was high (Case A3, Figure 4). We also used apparent spread *via* the CSF as an inclusion criterion, and accordingly, we had no cases of type III metastasis (a distant parenchymal nodule without connection to the CSF) (5).

## Methods

The inclusion criteria were MRI of a primary intra-axial lesion combined with lesions suggestive of CSF drop metastasis and histological confirmation of glioma. It was required that anatomically distinct lesions were histologically assessed (e.g., the primary mass at the time of surgery and non-contiguous CSF drop metastases at the time of necropsy). Medical records at the Purdue, Auburn, and Virginia–Maryland Colleges of Veterinary Medicine were searched for dogs meeting the inclusion criteria. Information retrieved from the medical record included signalment, neurolocalization, therapy received, the results of all surgical biopsy, necropsy, and histopathological examinations and the dates of all MRIs, therapies, and deaths. Glioma type (astrocytoma or oligodendroglioma) and grade (low or high) were classified according to recently revised canine-specific criteria (17).

MRI was reviewed. The MRI equipment, sequence, and imaging protocol used in each case are available in Supplementary File 1. Primary tumors were described according to canine glioma MRI criteria (1, 3). Metastatic lesions were classified according to a scheme for characterizing human glioma dissemination (leptomeningeal, ependymal, satellite, or mixed) (5). Post-treatment MRI lesions were classified using the human *updated Response Assessment in Neuro-Oncology* (9) as recommended for canine glioma (10): complete remission (elimination of all enhancing tumor), partial remission (≥50% decrease in enhancing tumor), or stable disease (<50% decrease or <25% increase in enhancing tumor). Progressive disease was designated if any of the following were present: ≥25% increase in enhancing tumor, increased T2/FLAIR lesion burden, or any new lesion (9, 10).

## Data Availability Statement

The original contributions presented in the study are included in the article/Supplementary Material, further inquiries can be directed to the corresponding author/s.

## Ethics Statement

Ethical review and approval was not required for the animal study because this was a retrospective study performed on information available in medical records. No animal procedures were performed. Written informed consent for participation was not obtained from the owners because no procedures were performed for this retrospective study. However, owners did provide signed consent for information about their animals to be used in research, at the time of veterinary care.

## Author Contributions

RB and JR designed the study. RB, AY, AC-G, and JR provided case materials. RB, AY, and JR collected the data and drafted the manuscript. MM provided the histology figure and legend. HH edited the MRI figures and legends. All authors edited the manuscript. All authors approved the final manuscript.

## Conflict of Interest

The authors declare that the research was conducted in the absence of any commercial or financial relationships that could be construed as a potential conflict of interest.
