# Supplementary material for: Cerebrospinal Fluid Drop Metastases of Canine Glioma: Magnetic Resonance Imaging Classification
Source: Front Vet Sci. 2021 May 3;8:650320. doi: 10.3389/fvets.2021.650320 (PMC8126621; doi:10.3389/fvets.2021.650320)
Supplement: Supplementary file 1 [file Data_Sheet_1.docx]

**Supplementary File 1. MRI equipment, sequences and imaging protocol**

*Purdue University College of Veterinary Medicine*

*Cases A1, A2 & B1*

1.5 T General Electric Signa, Milwaukee, WI.

Sequences performed included T2-weighted (sagittal, dorsal and transverse), T1-weighted before and after contrast administration (sagittal, dorsal and transverse), and FLAIR, GRE and DWI (transverse).

Repetition time and echo time: 617–800 and 12.8–19.2 ms (T1‐weighted), 3,000–5,500 and 101.8–118.7 ms (T2‐weighted), 8,002 and 123.6 ms (T2‐FLAIR), 400 and 15 ms (GRE) and 8,000 and 79.8 ms (DWI). Slice thickness was 1.5–2.1 mm. Field of view was 16–20 cm.

When spinal imaging was also performed, slice thickness was 2.3 mm (sagittal) or 3.0 (transverse). Field of view was 14–26 cm.

*Virginia-Maryland College of Veterinary Medicine*

*Cases A4 – A7 & B3*

1.5 T Philips Intera, Andover, MA.

Sequences performed included T2-weighted (sagittal and transverse), T1-weighted before and after contrast administration (sagittal, dorsal and transverse), and FLAIR, GRE and DWI (transverse).

Repetition time and echo time: 300-417 and 11.0 – 11.4 ms (T1-weighted), 2,418-3,800 and 100-105 ms (T2-weighted), 8,002-9,000 and 133-140 ms (T2-FLAIR), 600-970 and 15-23 ms (GRE) and 7,942-10,000 and 122-154 ms (DWI). Slice thickness was 4.0 mm. Field of view was 14-19 cm.

When spinal imaging was also performed, slice thickness was 3.0 mm (dorsal, sagittal and transverse). Field of view was 21-31 cm.

*Auburn University College of Veterinary Medicine*

*Case B2*

1.5 T Phillips Achieva, Andover, MA.

Sequences performed included T2-weighted (sagittal and transverse), T1-weighted before and after contrast administration (sagittal, dorsal and transverse), and FLAIR, GRE and DWI (transverse).

Repetition time and echo time: 600 and 10-11.5 ms (T1-weighted), 5,000 and 110-118 ms (T2-weighted), 8,000 and 150 ms (T2-FLAIR), 843 and 23 ms (GRE) and 7,211 and 143 ms (DWI). Slice thickness was 3.0 mm. Field of view was 21-23 cm.

When spinal imaging was also performed, slice thickness was 3.0 mm (sagittal, dorsal and transverse). Field of view was 18-30 cm.

*Referring Private Practice Hospital*

*Case A3*

0.3 T Hitachi AIRIS

Sequences performed included T2-weighted (sagittal and transverse), T1-weighted before and after contrast administration (sagittal, dorsal and transverse), and FLAIR (transverse and dorsal).

Repetition time and echo time: 385 and 9.3 ms (T1-weighted), 5,000 and 120 ms (T2-weighted), and 8,002-6,320 and 91 ms (T2-FLAIR). Slice thickness was 3.9 – 5.0 mm. Field of view was 17-23 cm.
